# Circular RNAs as novel potential biomarkers for pancreatic cancer

**DOI:** 10.7150/jca.58640

**Published:** 2021-06-01

**Authors:** Shanshan Liu, Qiuyue Li, Yan Ma, Christopher Corpe, Jin Wang

**Affiliations:** 1 Shanghai Public Health Clinical Center, Fudan University, 2901 Caolang Road, Jinshan District, Shanghai 201508, China.; 2 King's College London, London, Nutritional Science Department, 150 Stamford street, waterloo, London, SE19NH, United Kingdom.

**Keywords:** circRNAs, pancreatic cancer, biomarkers, diagnostics, therapy

## Abstract

Pancreatic cancer (PaCa) is the fourth leading cause of cancer-related deaths in the United States, and the vast majority of these malignancies are pancreatic ductal adenocarcinomas (PDAC), but there is still a lack of early detection biomarkers for PaCa. Unlike linear RNAs, circRNAs form covalently closed continuous loops and can act as mammalian gene regulators. They may be diagnostic or predictive biomarkers for some tumors, also be novel potential therapeutic targets in different diseases. This review focuses on (1) the biogenesis of circRNAs, RNA binding proteins (RBPs) and complementary sequences of circRNAs; (2) the characteristics of circRNAs which allow them to interact with miRNAs; (3) the roles of circRNAs playing in the regulation of gene expression, cell behavior and cancer, and their potential role as novel biomarkers and therapeutic targets in pancreatic cancer.

## Introduction

Pancreatic cancer (PaCa) is an aggressive malignancy characterized by strong invasion. PaCa is also difficult to cure and has a poor prognosis, seriously deteriorating the patients' quality of life 1. Due to the lack of effective biomarkers for the early diagnosis of this malignancy, patients often receive treatment when it is too late and the survival rate of patients diagnosed with PaCa after five years is < 6% 2. Recently, circular RNA (circRNAs) is becoming a new research hotspot in the field of RNAs. circRNAs are widely dispersed in eukaryotic cells, and enriched and stabilized in many tissues, manipulating gene expressions 3-5. circRNAs play a key role in the development and progression of human diseases and are involved in the proliferation, apoptosis, invasion and metastasis of various cancers 6, 7 including breast cancer (BCa) 8, colorectal cancer (CRC), gastrointestinal stromal tumor (GIST) 9, prostate cancer (PCa) 10, esophageal squamous cell carcinoma (ESCC) and pancreatic ductal adenocarcinoma (PDAC) 11-14. circRNAs are promising diagnostic or predictive biomarkers for certain diseases, and in particular early stage of PaCa. In this review, we hypothesize that circRNAs may serve as targets for the development of early biomarkers of PaCa, which is significant for the early diagnosis of PaCa.

## circRNAs biogenesis and regulatory mechanisms

### Biogenesis of circRNAs, RNA binding proteins (RBPs) and complementary sequences

**Figure 1:**
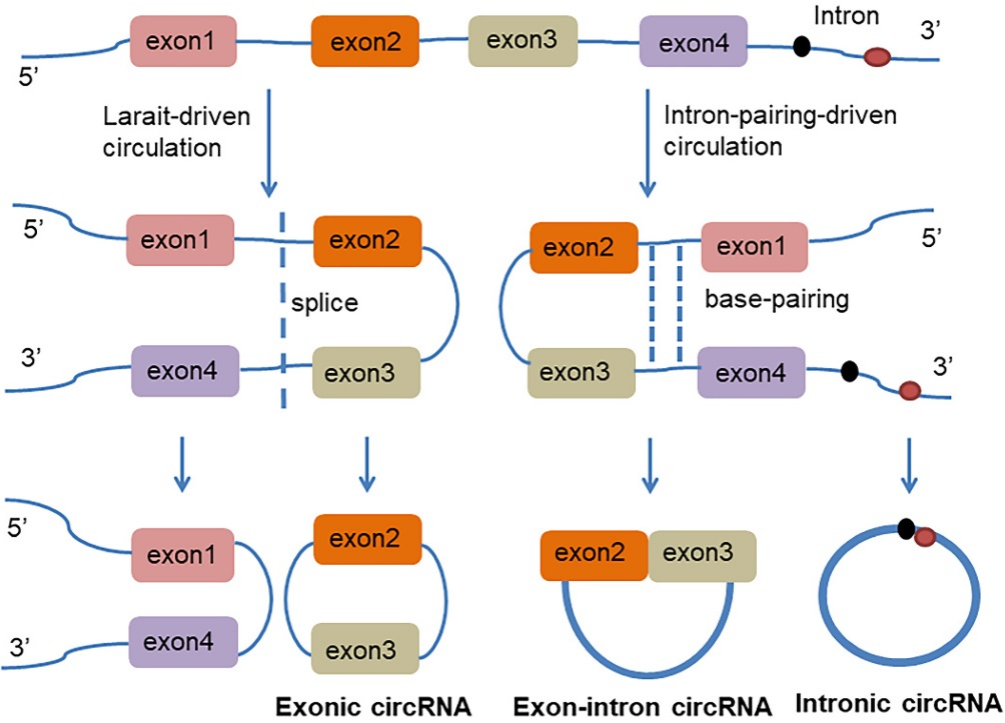
The generation of Exon-intron circRNA and intronic circRNA. The mechanism by which circRNAs are formed is classified as model 1 "lasso-driven circularization" or "exon jump" (The generation of extronic circRNA); model 2 "intron-pair-driven circularization" or "direct back-stitching".

circRNA is a class of non-coding RNAs that are produced in the nucleus and are ubiquitous in eukaryotic cells. They are also characterized by a covalently closed continuous loop without 5' or 3' polarities structure. Because circRNAs are easily degraded by ribonuclease, they can be stably expressed in the cytoplasm. In some cases, the expression levels of circRNAs are tenfold greater than linear RNA and have a rich genetic diversity 15. Most of them are transcribed from protein-coding genes by RNA polymerase II 16, 17. Precursor messenger RNA (mRNA) containing exons and introns are produced in the nucleus, and then the pre-mRNA is transferred to the cytoplasm, which is cleaved into introns or exons. However, due to splicing diversity, circRNAs are mainly produced by rearrangement of exons. The recovery process involves RNA cyclization, which is facilitated by a covalent linkage between the downstream splice donor site (50 splice sites) and the upstream receptor splice site (30 splice sites). Therefore, circRNAs can be formed by lasso-driven circularization or exon jump model, intron-pair-driven circularization or direct back-stitching model 15, as shown in **Figure 1**. Intron pair-driven cyclization may be more frequent than lasso-driven cyclization and invert complement sequences 18 such as the inverted repeat Alu pair (IRAlus), which is an important sequence pair for circRNAs biogenesis 16, 19-25. circRNA also has a high degree of conservation of reverse splicing, although circRNA is classified into intron circRNA and exon circRNA. The intron circRNA is composed of a 2'-5' chain, and the exon circRNA is composed of a 3'-5' chain without a 2'-5' chain 26.

Reverse complementary sequences or RNA binding proteins (RBPs) are also required for the formation of circRNAs 27. circRNAs also may act as protein sponges, by binding RNA-binding proteins (RBPs) which can act as activators or inhibitors of circRNAs formation 16, 28. Muscle blind protein (MBL) can strongly and specifically bind to the circRNAs which are generated from its own RNA. The RNA sequence between the MBL dimers forms a lariat structure, which allows the receptor and the donor to be spatially close to each other; thereby inducing RNA reverse splicing, hindering linear splicing, and stimulating circMBL production 16. RNA pairing competition between or within a single flanking intron may significantly impact splicing selection and result in the processing of multiple circRNAs transcripts from a single gene by complementary sequences (repeats or non-repetitive sequences) which may be beneficial for exon cyclization. Exon circularization can also be mediated by complementary sequences in human introns 20.

### circRNA interacts with miRNAs as miRNAs sponges

circRNAs are rich in miRNA binding sites and function as miRNA sponges, which can regulate the production of intracellular proteins. Competing endogenous RNAs (ceRNAs) regulate other RNA transcripts including mRNAs, long non-coding RNAs (lncRNAs) and pseudogenes by battling with shared microRNAs (miRNAs) 29. circRNAs may have one or more miRNA response elements (MREs) 30, 31 which can bind to the corresponding miRNAs and induce the expression recovery of genes downstream of miRNAs, so that circRNA has the ability to bind to miRNAs, influencing miRNAs function and targets 31-33. Thus, the presence or absence of ceRNA affects the ability of miRNAs to regulate gene expression, suggesting circRNAs could act as miRNAs sponges or potent ceRNA molecules, and could deplete polymorphisms at miRNAs binding sites 13, 31, 33-35.

To date, circRNAs sponges are characterized by high expression levels and a large number of miRNAs binding sites. ciRS-7/CDR1as (a circular RNA sponge for miR-7 or CDR1 antisense) and SRY (the sex determining region Y) can be used as miRNAs sponges 12, 19, 33. The brain degeneration-associated protein 1 (CDR1) gene can be translated into a natural circular antisense transcript, known as the antisense of the cerebellar degeneration-associated protein 1 transcript (CDR1as) 36. SRY is carried out by long inverted repeats (IRs) of more than 15.5 kb in length. When one or two IRs are deleted, no cyclicization occurs 19. The SRY gene can mediate the sex of a mammal and is highly expressed in the mouse testis and produces a circRNA containing 16 miR-138 binding sites, which acts as a miR-138 sponge 33, 37. CDR1 is highly expressed in the brain and contains at least 60 miR-7 binding sites, overwhelming any known linear sponge 31, 33. miR-7 has a wide range of functions and is involved in a variety of signaling pathways. ciRS-7 is also highly associated with Argonaute (AGO) protein in a miR-7-dependent manner and many tumors can be regulated by the ciRS-7-miR-7 axis 38. Although circRNAs are fully resistant to miRNAs-mediated targeted destabilization, they strongly inhibit miR-7 activity, leading to elevated levels of miR-7 targets 33. Thus, miR-7 can bind tightly to ciRS-7, and regulation of the miR-7/miR-671/ciRS-7 axis may play an important role in cancer-associated biological processes.

More importantly, the role of miR-7 and SRY in the development and progression of cancer has been demonstrated, suggesting that circRNAs may mediate physiological and pathological processes through binding to miRNAs 39. Hence, circRNA sponges may have more research value than linear sponges, which not only can be involved in gene regulation but also play an important role in cancer. Our functional network analysis also showed that circ_0057558 and circ_0034467 regulated miR-6884, and circ_0062019 and circ_0060325 regulated miR-5008 as the key regulators of PCa 10.

### circRNAs may regulate host gene expression

There are three mechanisms by which circRNA regulates parental gene expression. Firstly, circular intronic RNAs (ciRNAs) are produced by introns and bind to RNA polymerase II (Pol II) and promote transcription 40. Secondly, exon-intron circRNAs (EIciRNA) bind to U1 snRNP (U1 small nuclear ribonucleoproteins) forming a EIciRNA-U1 snRNP complex that may interact with the RNA Pol II transcription complex to promote host gene expression 41. Finally, circRNA acts as a miRNA sponge that increases transcript translation of its parent gene 13. The formation of circRNAs depends on the critical flanking RNA elements that might be required for introns 42. These circRNAs have little enrichment for miRNA target sites, indicating that they are functionally different 42. Detailed studies have shown that some ciRNAs are prevalent in the nucleus and interact with Pol II mechanism and regulate the host transcriptional activity in a cis manner 26. For example, EIciRNAs such as circEIF3J and circPAIP2 involved in RNA Pol II are mainly situated in the nucleus, interact with the U1-snRNPs and enhance their transcriptional parental genes in a cis-acting manner 43. In short, circRNAs enhance the ability of transcription and translation by regulating parent genes.

### circRNAs bind to proteins and regulate cell behavior

circRNA can also encode a variety of proteins with different functions. Synthetic circRNAs contain an inner chromosomal entry site (IRES) that can be efficiently translated into proteins 44. circAmotl1 can bind to PDK1 and AKT1 in cardiomyocytes, resulting in AKT1 phosphorylation and transportation into the nucleus to protect the myocardium from damage 16. It can also bind to STAT3 (signal transduction and transcriptional activator 3) and c-Myc which then transfer to the nucleus to promote cell proliferation, invasion and tumorigenesis 45, so that circAmotl1 may be used as a target for therapy for cancer.

## Deregulated circRNAs are associated with cancer

A large number of circRNAs have been identified to play key regulating roles in major tumor diseases, which have enlarged the regulatory networks of ceRNAs and provided a new direction for our in-depth study of the pathogenesis and human malignancies which are involved in the regulation of tumor cell production and growth in a variety of cancers. For example, in hypoxia-induced human umbilical vein endothelial cells (HUVEC) circ_0010729 was co-expressed with hypoxia-inducible factor 1α (HIF-1α) and negatively correlated with miR-186 46, revealing that the key regulation of circ_0010729 on vascular endothelial cell proliferation and apoptosis was determined by targeting the miR-186/HIF-1α axis. Esophageal cancer is the 8th most common cancer, one of which is ESCC. circITCH expression is usually down-regulated in ESCC, and circITCH might impose anti-tumor function in ESCC 13. Several studies have shown that miR-7 is lowly expressed in many types of tumors and negatively correlated with tumor growth and invasion 47-49. circITCH acts by interaction with miRNAs such as miR-7, with increased levels of miR-17 and miR-214 and ITCH, which promotes protein-mediated degradation of Dvl2 and reduces expression of the oncogene c-Myc. Thus, this process inhibits classical Wnt signaling and can prevent esophageal tumorigenesis 13. Based on the latest research, it was also found that the expression of circ_0067934 in ESCC was significantly higher than that in normal tissues. circ_0067934 is located in the chromosomal region 3q26.2, which consists of two exons joined by reverse splicing 50. Notably, circ_0067934 expression was also found to be involved in ESCC differentiation, T stage and TNM stage, as well as tumors with a lower degree of differentiation, higher expression levels of circ_0067934. In addition, circ_0067934 can promote ESCC cell proliferation and migration *in vitro*, and si-circ_0067934 can block the cell cycle of G2 phase.

We have analyzed the profiles of differentially expressed circRNAs in BCa 8, PCa 10 and GIST 9 by competing in endogenous RNAs microarray. A total of 4,370, 1,021, and 5,770 differentially expressed circRNAs were found in BCa, PCa and GISTs tumor tissue. Eight circRNAs, such as circ_0069094, circ_0062558, circ_0074026, circ_0079876, circ_0017536, circ_0023302, circ_0017650, and circ_0017545 were verified differentially expressed in BCa tissue and associated with TNM stage, lymph node infiltration, and Ki67 8. The expression levels of circ_0057558 and circ_0062019 in PCa tumor tissues were validated by qRT-PCR analysis 10. circ_0069765, circ_0084097, and circ_0079471 were also verified differentially expressed in 68 pairs of GISTs, comparing with the adjacent normal gastrointestinal tissues by qRT-PCR 9. We further demonstrated that the specific KIT-related regulation networks involved these three circRNAs, their host genes and miR-142-5p, miR-144-3p and miR-485-3p, which may be key regulators of GISTs 9.

circRNAs are also associated with hepatocellular carcinoma (HCC). circMTO1 acts as a sponge of miR-9, inhibits the progression of liver cancer, reduces the expression of circMTO1, and implies poor survival prognosis 3. The expression of circ_0001649 in HCC was lower than that in adjacent tissues, and its expression was related to tumor size and tumor embolus. It is worth noting that the larger the tumor size, the lower the expression of circ_0001649. Therefore, circ_0001649 might be involved in tumor growth and metastasis 5. High-throughput circRNA microarrays showed circ_0000520, circ_0005075 and circ_0066444 were significantly and abnormally expressed in HCC tissues 51. At the same time, ciRS-7 expression was significantly associated with liver MVI in serum AFP and HCC patients. Furthermore, ciRS-7 expression together with MVI expression was negatively correlated with miR-7 and synergistically associated with PIK3CD and p70S6K, which are targets of miR-7. Therefore, it means that ciRS-7 has become a new biomarker of liver MVI 52. circ_0007534 can bind to miR-761 and increase ZIC5 expression to promote the progression of glioma cells 53. In colorectal cancer, small interfering RNA (siRNA) knockdown of circ_0007534 significantly attenuated SW-620 and LoVo cell proliferation and promoted apoptosis. Tumor stage and lymph node metastasis are two diagnostic factors closely related to the expression of circ_0007534 54. Coincidentally, circ_0014130 (circPIP5K1A) is also involved in tumorigenesis in non-small cell lung cancer (NSCLC) 55. circ_0014130 can act as a miR-600 sponge to promote NSCLC proliferation and metastasis by promoting HIF-1α, suggesting circ_0014130 could be a novel candidate therapeutic target for NSCLC through the circ_0014130/miR-600/HIF-1 alpha axis. Through the circRNA-miRNA-mRNA axis, circRNA can upregulate or downregulate gene expression and affect tumor progression. Emerging evidence showed that circ_103809 was involved in the development of colorectal, lung and liver cancer through the circRNA-miRNA axis by participating in the proliferation and migration of cancer cells, and could thus provide new ideas for clinical treatment of cancer 56-58.

## circRNAs play key roles in pancreatic cancer

**Figure 2:**
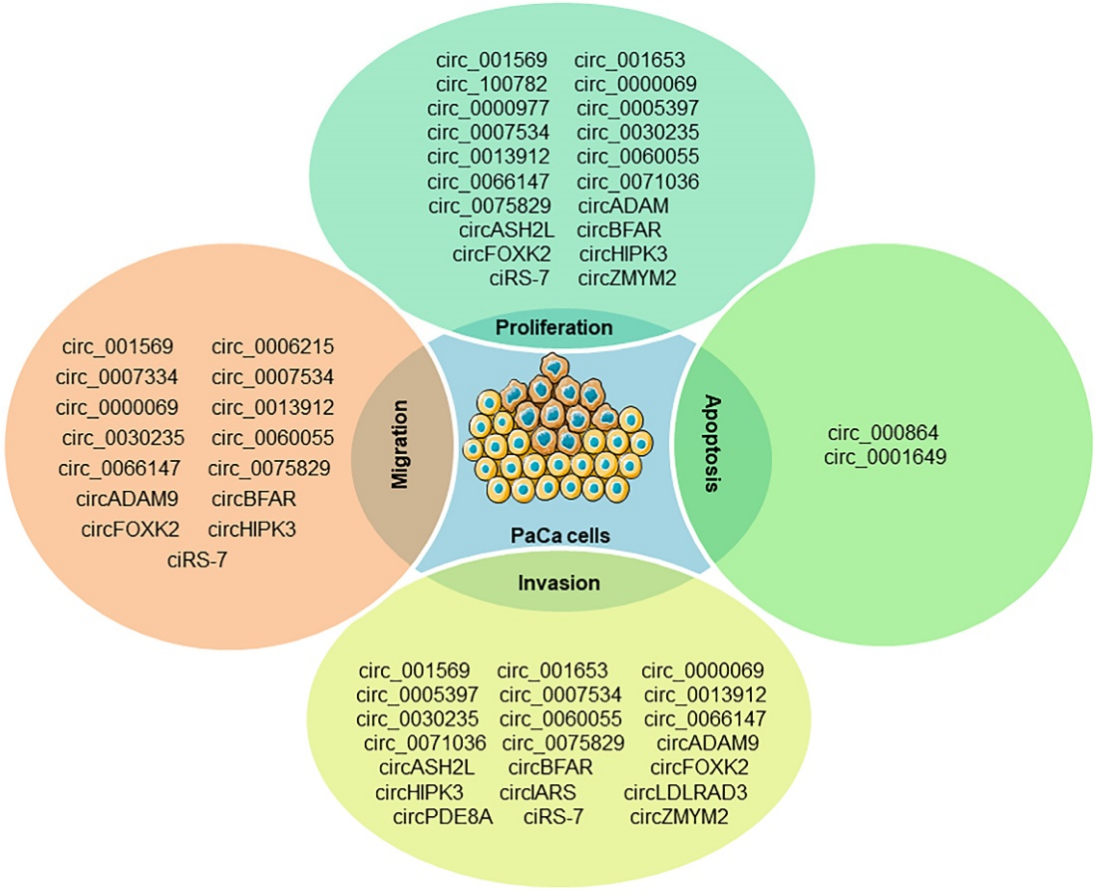
The known roles of circRNAs in PaCa progression.

**Figure 3:**
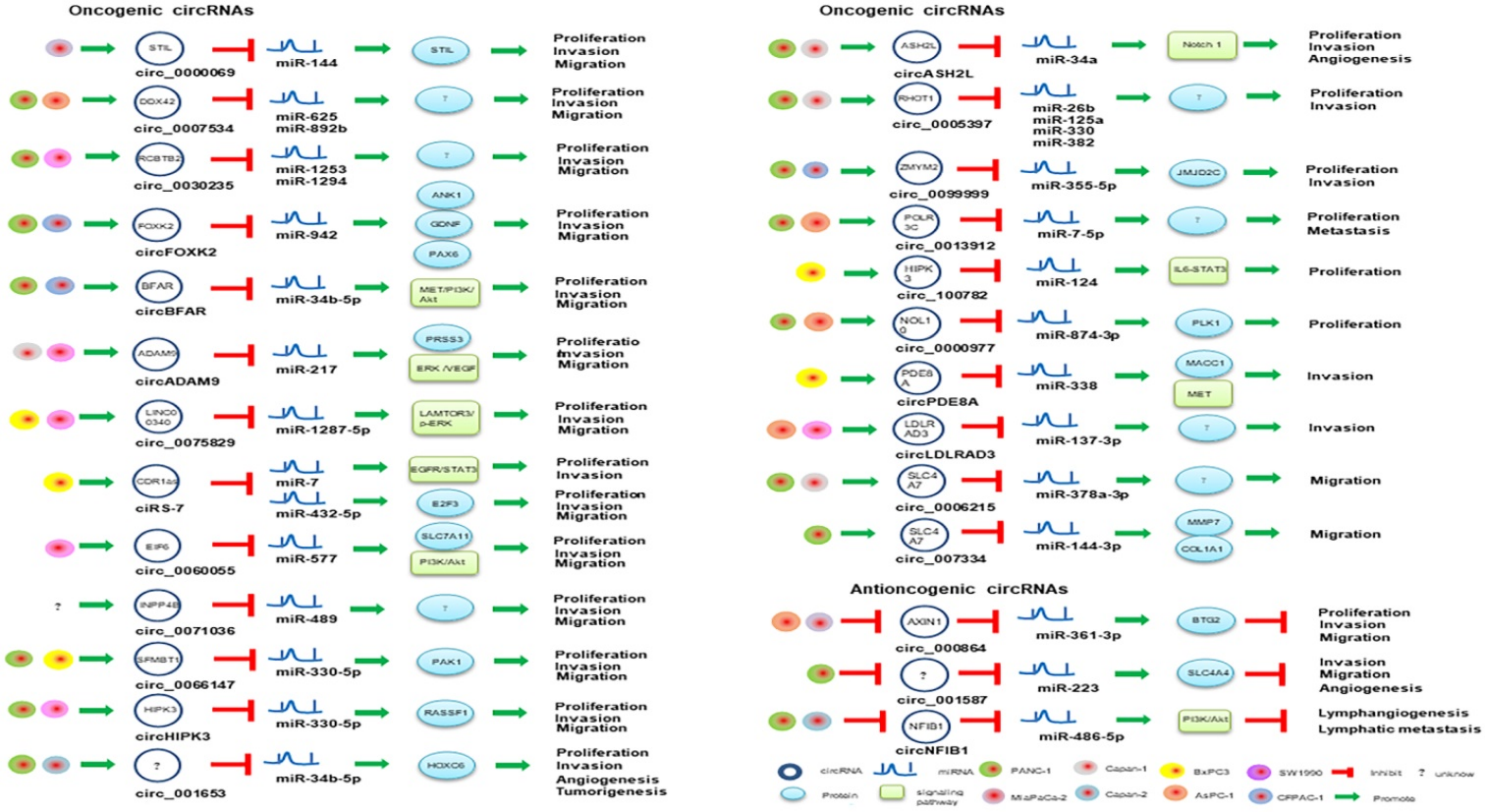
circRNAs involved in miRNA-associated gene regulatory pathway in PaCa.

PaCa is one of the most fatal malignancies in the digestive system. Understanding the molecular mechanisms underlying the initiation and progression of pancreatic cancer may promote the development of diagnostic and therapeutic strategies. miRNAs and circRNAs have been identified as important regulators of human cancer development 59, 60. A comprehensive literature search was executed in PubMed using the medical subject headings (MeSH) terms “pancreatic cancer” and “circRNA”, there are a total of 129 relevant studies for PaCa involved in circRNAs and only five review articles for pancreatic cancer found in our initial study, which revealed that circRNAs could serve as diagnostic, therapeutic or prognostic biomarkers for PaCa 61-65. Several oncogenic and antioncogenic circRNAs (circ_000864, circ_001587 and circNFIB1) have been discovered to regulate the proliferation, migration, invasion and angiogenesis of PaCa cells (**Figure 2**). Most of them could regulate PaCa-related signaling pathways including through miRNA sponges as shown in **Figure 3**.

### Oncogenic circRNAs in PaCa

circRHOT1 (circ_0005397) is produced by the sequence located at chr17: 30500849-30503232, and the splicing sequence is 233 nt in length. circRHOT1 might bind to miR-26b, miR-125a, miR-330 and miR-382 and regulate a variety of tumor-associated pathways 66. circRHOT1 was upregulated in PaCa tissues and cell lines, and silencing circRHOT1 could inhibit pancreatic cell proliferation, invasion and migration 66, which demonstrated that circRHOT1 might play an important role in pancreatic cancer through spongiform tumor-associated miRNAs as a novel potential therapeutic target for PaCa. The expression of circLDLRAD3 was significantly upregulated in PaCa tissue and PaCa plasma samples. circLDLRAD3 expression was associated with lymphatic invasion, venous invasion and metastasis 67. Functional studies of circLDLRAD3 may also improve our understanding of the pathogenesis of PaCa 67. Therefore, circLDLRAD3 has the potential to be a novel indicative biomarker for tumor invasion in the diagnosis of PaCa.

IL6-JAK2-STAT3 signaling pathway is activated in pancreatic cell lines and PDAC tissue samples 68. Activation of STAT3 is closely related to tumorigenesis, and STAT3 can cause tumor cell proliferation and migration 69. circ_100782 plays a vital role in PDAC. Chen *et al.* revealed that circ_100782 was significantly upregulated in pancreatic cancer. Silencing circ_100782 through the sponge effect of circRNAs regulates the STAT3 pathway by targeting miR-124, which subsequently inhibits the proliferation of pancreatic cancer cell BxPC-3. Thus, circ_100782 plays an important role in the pathogenesis of PaCa.

circCDR1as not only promotes the migration, invasion and proliferation of PaCa cells via regulating E2F3 expression by sponging miR-432-5p 70, but mediates EGFR/STAT3 signaling pathway as a sponge of miR-7 71. circ_0000977 was abnormally upregulated in pancreatic cancer, and silencing circ_0000977 inhibited pancreatic cancer cell proliferation and induced cell cycle arrest by stimulating miR-874-3p and inhibiting PLK1 expression 72. circ_0007534 participates in the development of colorectal cancer and glioma, and the formation of PDAC 73. circ_0007534 was upregulated in PDAC tissues and PANC-1, SW1990 and BxPC-3 cells, accelerated cell proliferation, migration and invasion, inhibited cell apoptosis, and was positive correlation with poor PDAC phenotype 73. Previously, miR-625 and miR-892b were identified as tumor suppressor molecules in cancer. Hao *et al.* confirmed that circ_0007534 can directly interact with miR-625 and miR-892b 73, which implied that circ_0007534/miR-625/miR-892b regulation axis might contribute to the development of PDAC. circ_0013912, circ_0000069, circ_0071036, circ_0075829, circ_0099999 could also facilitate the proliferative, migratory and invasive rates of PaCa cells through sponging miR-7-5p, miR-144, miR-489, miR-1287-5p and miR-335-5p, respectively 74-78. circSFMBT1 serves as a miR-330-5p sponge and also promotes PaCa growth and metastasis via regulating miR-330-5p/ PAK1 axis 79. Silencing of circ_001653 in PDAC cells can inhibit cell proliferation, cell-cycle progression, angiogenesis, and invasive properties by binding to miR-377 80. Besides, circBFAR, circASH2L, circFOXK2, circADAM9, circEIF6 and circRNA chr7:154954255-154998784+ were significantly up-regulated in PDAC by sponging miR-34b-5p, miR-34a, miR-942, miR-217, miR-557 and miR-4459, respectively 81-86, which play key roles in tumor invasion and might therefore be useful diagnosis biomarkers of PDAC.

### Antioncogenic circRNAs in PaCa

Several antioncogenic circRNAs such as circ_000864, circ_001587, circ_0001649 and circNFIB1 were repressed in PaCa tumor or cancer cells 87-90. circ_000864 was downregulated in AsPC-1 and MiaPaCa-2 cells, and it could upregulate BTG2 expression and inhibited the proliferation, invasion and migration of PaCa cells as miR-361-3p sponges 87. circ_001587 mediated miR-223/SLC4A4 axis and also could inhibit PaCa cell migration, invasion and angiogenesis 88. Besides, circNFIB1 was suppressed in PANC-1 and Capan-2 cells and regulated PI3K/Akt signaling pathway by attenuating the oncogenic effect of miR-486-5p, which was negatively associated with lymph node metastasis in PaCa patients 89. circ_0001649 was also downregulated in PaCa tissues and cell lines, and its low expression has been associated with advanced tumor stage and poor tissue grade. Exogenous administration of circ_0001649 can inhibit the proliferative ability of PaCa cell lines and induce apoptosis, suggesting it could be used as an exogenous anticancer agent 90.

### circRNAs were associated with gemcitabine (GEM) resistance in PaCa

CircHIPK3 as a sponge for miR-330-5p which directly bounds to the 3' UTR of RASSF1 could promote cell proliferation, invasive, migration and EMT, which was associated with GEM resistance in PaCa cells 91. Ding *et al.* showed the expression profiles of circRNAs in gemcitabine-resistant PaCa cell lines were clearly different to normal PaCa cell lines. They also observed that the differentially expressed circRNAs played a role in chemoresistance in PaCa by acting as a miRNA sponge that affected MRPK and mTOR signaling pathways 92. Huang *et al.* analyzed circRNA expression profiles in PANC-1-GR (gemcitabine resistant cell line) and PANC-1, and found two circRNAs (chr14:101402109-101464448+, chr4:52729603-52780244+) to be significantly different. They also found that silencing the expression of the genes restored the sensitivity of PaCa resistant cell lines to gemcitabine while their overexpression weakened this sensitivity 93.

### circRNAs were involved in protein translation

circRNAs not only play a role in gene transcription, but they can be involved in protein translation. Studies have shown that N^6^-methyladenosine (m^6^A) is the most ubiquitous base modification of RNA, and a single m^6^A site is sufficient to drive translation initiation, which can promote the efficient initiation of protein translation of circRNA in human cells. The methylation of circRNAs synergizes with IRES to improve the efficiency of circRNAs in translating proteins. The *p16* gene is involved in cell cycle regulation and tumor suppressor processes, and its inactivation mechanisms include deletions, mutations, and aberrant methylation of 5'-CpG islands. Abnormal methylation of 5'-CpG islands has been found in a variety of tumors as the primary mechanism for their inactivation 94. Translation of circRNAs may be more prevalent in cancer cells because circRNAs contain large amounts of m^6^A modifications sufficient to drive protein translation in a cap-independent manner to promote cancer cell development, apoptosis and cell cycle regulation 95. circZNF609 contains an open reading frame and is translated into protein in a cap-free manner, which can specifically control the proliferation of myoblast 96. Moreover, circ-ZNF609 acts as a competitive endogenous RNA, regulating AKT3 expression by a sponge miR-150-5p in Hirschsprung disease 97. circZNF609 can also induce the expression of cancer-related proteins, regulate cell proliferation, and participate in the tumor suppressor process in PaCa.

### circRNAs and exosomal circRNAs as diagnostic and prognostic biomarkers for pancreatic cancer

Wu *et al*. found that three circRNAs (circ_004183, circ_079265 and circ_105039) were downregulated in plasma from children with CHD (congenital heart diseases), suggesting they may be crucial in the development of CHD and serve as novel non-invasive biomarkers for the diagnosis of CHD in children 98. circRNAs are frequently upregulated in gastric cancer (GC) tissues and promote cell growth through members of the spongy miR-125 family, and as such have been described as potential disease biomarkers in human saliva 99. circ_002059 was downregulated in the GC and could represent a potential novel biomarker for GC diagnosis 100-102. Combination of circ_0057558, circ_0062019 and PSA level showed significantly increased AUC, sensitivity and specificity of PCa than PSA alone 10. We also found that circ_0069094, circ_0079876, circ_0017650, and circ_0017526 were upregulated in the plasma of those patients with BCa, in contrast to normal controls 8, which suggested that plasma circRNAs might be potential biomarkers for cancer. In addition, circ_0007334 promoted the expression levels of matrix metallopeptidase 7 (MMP7) and collagen type I alpha1 chain (COL1A1) by blocking the functions of miR-144-3p and miR-577 in PDAC 103 and may be used as a potential biomarker of diagnosis and therapy for PDAC. Furthermore, the therapeutic value of circRNAs as biomarkers has been explored in many studies. circIARS, ciRS-7 and circLDLRAD3 were involved in vascular or lymph node invasion 67, 71, 104. circIARS, circPDE8A, circ_0001649, circ_0007534, circ_001569 and circ_0030235 could be as a classifier with TNM stages to evaluate the risk of recurrence 73, 90, 104-107. Therefore, these circRNAs may be correlated with cancer progression and could be a novel diagnostic and prognostic markers for PaCa (**Table 1**).

**Figure 4:**
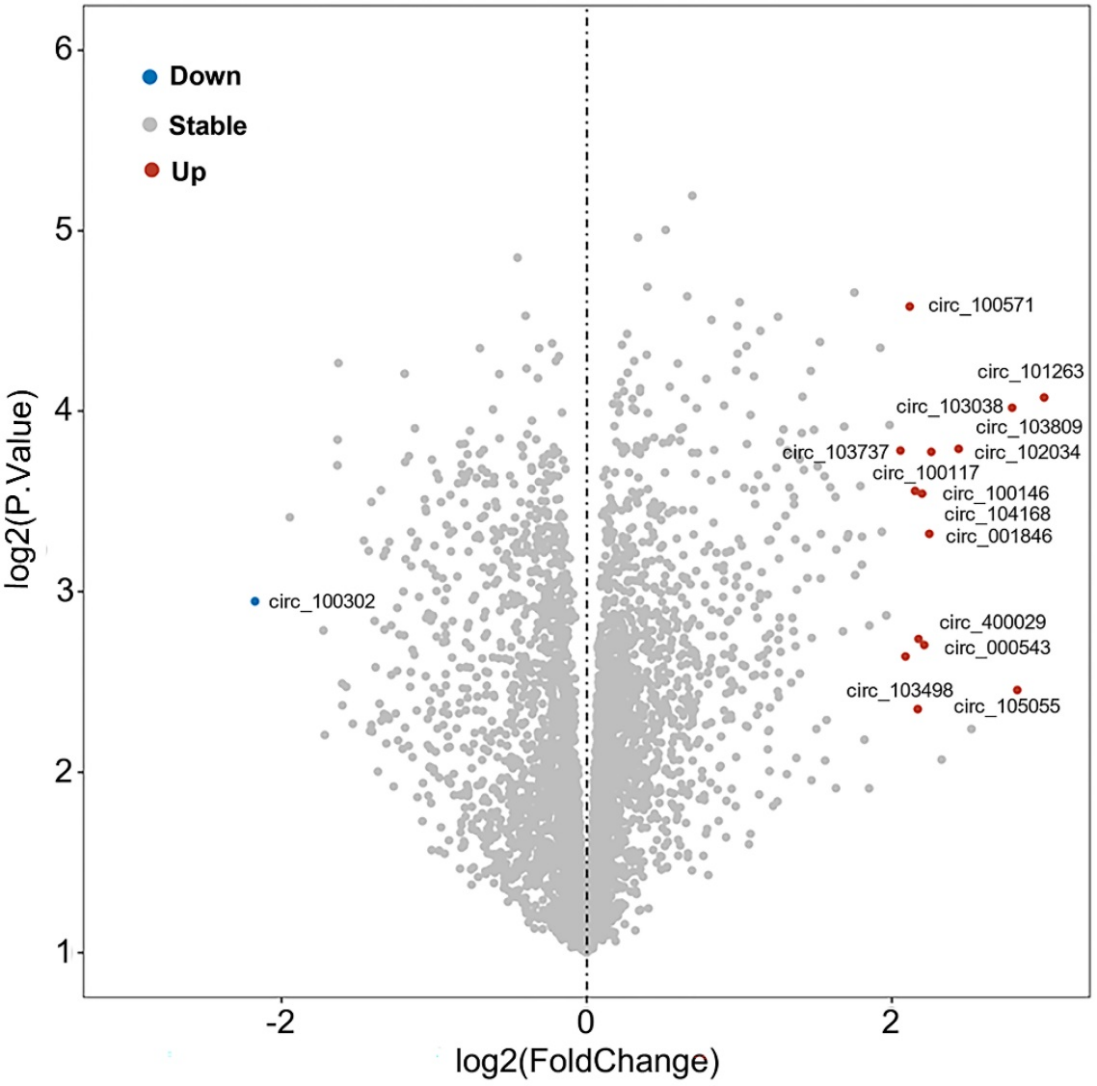
Volcano analysis of differentially expressed circRNAs in pancreatic tumor from GEO (No. GSE69362).

Further, the expression profile of circRNAs in PDAC has been analyzed in adjacent tissues of 6 pairs of PDAC patients using microarray technology 108. Comparing normal tissues with pancreatic tumor tissues, it was shown that sixteen circRNAs were significantly deregulated, specifically fifteen circRNAs were upregulated and one circRNA (circ_100302) was repressed, which were recorded in Gene Expression Omnibus (GEO; No. GSE69362) 108 (**Figure 4**, **Table 2**). Zhao *et al* also analyzed the circRNA expression profiles of pancreatic cancer from GSE79634 and GSE69362 datasets and constructed a ceRNA network which was involved in the NF-kappa B, PI3K-Akt, and Wnt signaling pathways 109. Thus, we speculated that these differentially expressed circRNAs were critical to PaCa progression and could be developed as a novel biomarker, therapeutic targets for PaCa need further exploration.

Exosomes are critical mediators of intercellular communication that can regulate a diverse range of biological processes between cells 110. Exosomal circRNAs can originate from tumor cells, or other cells, such as activated human platelets and adipose cells, and enriched in the circulation and urine 111, can transfer biological information to specific cells, and might stimulate cancer 112. Exosomal circRNAs have also been found in platelet-derived extracellular vesicles 113, PaCa 105, and cholangiocarcinoma 114. Recent RNA sequencing data indicated that many circRNAs are stable and enriched in exosomes and could be a promising biomarker for cancer diagnosis. Exosomal circPDE8A can act as a ceRNA for miR-338 to regulate MACC1 and promote invasive growth via the miR-338/MACC1/MET pathway in PaCa 105 (**Figure 3**). Also, exosomal circPDE8A could be detected in blood circulation and correlated with progression and prognosis in PaCa patients. Thus, exosomal circRNAs would be potential therapeutic targets. Exosomal circRNAs are expected to become remarkable biomarkers and therapy tools for PaCa.

## Summary and prospect for circular RNAs

In recent years, as the number of circRNAs has increased, the functions of circRNAs have extended enormously. As a large class of RNA with an extensive ability to regulate genes 31, circRNAs are more stable and highly conserved than linear mRNAs. As a sponge for miRNAs, circRNAs can bind miRNAs to regulate transcription or influence parental gene expression, and mediate the entire process of physiology and pathology. circRNAs can also regulate the production of intracellular proteins, regulate cell behavior and participate in defense mechanisms *in vivo*. In terms of tumor formation, circRNAs can further regulate tumorigenesis, participate in tumor formation, metastasis, invasion, and cancer-related pathways through the circRNA-miRNA axis. The roles of circRNAs in tumor immunity, extracellular transfer, involvement in protein regulation and their transformation into functional proteins can also affect tumor progression. Considered as novel biomarkers in cancer development and progression, circRNAs have been used in tumor targeted therapies.

Since the pathological and biological characteristics of PDAC lead to the lack of specificity of early symptoms 115, the heterogeneity of PaCa makes it difficult to cure. Although the value of circRNAs in PDAC has been gradually identified, there are still some challenges in early diagnosis of PDAC. Based on the current research of circRNAs, it is believed that circRNAs can be used as novel biomarkers for the diagnosis of pancreatic cancer, solving the problem of early diagnosis of pancreatic cancer, and providing a new therapeutic target for the treatment of this malignancy.

## Figures and Tables

**Table 1:** Clinical significance of circRNAs for PaCa

circRNA ID	Host gene	Patients (n)	Expression (High/Low)	Differentiation (Well/Poorly)	Clinicopathological association	Biomarker type	Ref.
circ_0001649	SHPRH	58	25 /33	31 /27	T stage	Prognostic biomarker	90
circ_001569	IRF4	26	13 /13	19 /7	T stage	Diagnostic and prognostic marker	107
circ_0007534	DDX42	60	30 /30	34 /26	T stage, lymph node invasion	Prognostic biomarker	73
circ_0030235	RCBTB2	62	32 /30	34 /28	T stage, lymph node invasion	Prognostic biomarker	106
circASH2L	ASH2L	90	45 /45	9 /66	Lymphatic invasion, TNM stage	Diagnostic marker	82
circIARS	IARS	85	42 /43	65 /20	Liver metastasis, vascular invasion, TNM stage	Diagnostic and prognostic marker	104
circLDLRAD3	LDLRAD3	30	18 /12	-	Venous invasion, lymph node invasion	Diagnostic marker	67
circPDE8A	PDE8A	93	46 /47	72 /21	Lymphatic invasion, TNM stage	Diagnostic and prognostic marker	105
circZMYM2	ZMYM2	106	73 /33	88 /18	-	-	74
ciRS-7	CDR1as	41	-	-	Lymph node metastasis, venous invasion	-	71

**Table 2:** Deregulated circRNAs in pancreatic tumor tissue

circRNAs ID	Alias in circBase	Host gene	Length	Strand	Position	Expression	Ref.
circ_100571	circ_0018004	PDSS1	195	+	chr10:27024168-27024508	Upregulated in PaCa tumor tissue	108
circ_101263	circ_0030235	RCBTB2	318	-	chr13:49075877-49077050	Upregulated in PaCa tumor tissue	108
circ_103038	circ_0060055	EIF6	906	-	chr20:33866724-33872064	Upregulated in PaCa tumor tissue	108
circ_103809	circ_0072088	ZFR	693	-	chr5:32379220-32388780	Upregulated in PaCa tumor tissue	108
circ_102034	circ_0005397	RHOT1	233	+	chr17:30500849-30503232	Upregulated in PaCa tumor tissue	108
circ_103737	circ_0070934	LARP1B	745	+	chr4:128995614-129012667	Upregulated in PaCa tumor tissue	108
circ_100117	circ_0007895	EYA3	429	-	chr1:28362054-28384605	Upregulated in PaCa tumor tissue	108
circ_100146	circ_0011385	EIF3I	278	+	chr1:32691771-32692131	Upregulated in PaCa tumor tissue	108
circ_104168	circ_0008514	RTN4IP1	463	-	chr6:107031202-107050797	Upregulated in PaCa tumor tissue	108
circ_001846	circ_0000520	RPPH1	123	-	chr14:20811436-20811559	Upregulated in PaCa tumor tissue	108
circ_400029	circ_0092337	RPL13	360	+	chr16:89628179-89628539	Upregulated in PaCa tumor tissue	108
circ_000543	circ_0000326	TCONS_l2_00004572	96	+	chr11:65272490-65272586	Upregulated in PaCa tumor tissue	108
circ_400068	circ_0092314	RANBP1	340	+	chr22:20113099-20113439	Upregulated in PaCa tumor tissue	108
circ_105055	circ_0001946	CDR1	1485	+	chrX:139865339-139866824	Upregulated in PaCa tumor tissue	108
circ_103468	circ_0067260	COPG	169	+	chr3:128973510-128973920	Upregulated in PaCa tumor tissue	108
circ_100302	circ_0013587	LRIG2	291	+	chr1:113661854-113662145	Downregulated in PaCa tumor tissue	108

## Abbreviations

AGO: Argonaute
BC: breast cancer
CDR1as: antisense of the cerebellar degeneration-associated protein 1 transcript
CHD: congenital heart diseases
ceRNAs: Competing endogenous RNAs
ciRNAs: circular intronic RNAs
circRNAs: circular RNAs
CRC: colorectal cancer
COL1A1: collagen type I alpha1 chain
ESCC: esophageal squamous cell carcinoma
EIciRNA: exon-intron circRNAs
GC: gastric cancer
GEO: Gene Expression Omnibus
GIST: gastrointestinal stromal tumor
HCC: hepatocellular carcinoma
HIF-1α: hypoxia-inducible factor 1α
HUVEC: human umbilical vein endothelial cells
lncRNAs: long non-coding RNAs
IRAlus: inverted repeat Alu pair
IRES: inner chromosomal entry site
IRs: inverted repeats
m^6^A: N^6^-methyladenosine
MBL: Muscleblind protein
microRNAs: miRNAs
MREs: miRNA response elements
MMP7: matrix metallopeptidase 7
mRNA: messenger RNA
PaCa: Pancreatic cancer
NSCLC: non-small cell lung cancer
PDAC: pancreatic ductal adenocarcinomas
PCa: prostate cancer
RBPs: RNA binding proteins
Pol II: RNA polymerase II
siRNA: small interfering RNA
SRY: sex determining region Y
STAT3: signal transduction and transcriptional activator 3
U1 snRNP: U1 small nuclear ribonucleoproteins
